# Implementing Bundle Care in Major Abdominal Emergency Surgery: Long-Term Mortality and Comprehensive Complication Index

**DOI:** 10.1007/s00268-022-06763-y

**Published:** 2022-09-28

**Authors:** Rune Munch Trangbæk, Jakob Burcharth, Ismail Gögenur

**Affiliations:** 1 Department of Surgery, Slagelse Hospital, Fælledvej 11, 4200 Slagelse, Denmark; 2 Department of Surgery, Zealand University Hospital, Køge, Denmark

## Abstract

**Background:**

Major abdominal emergency surgery (MAES) has a high risk of postoperative mortality and a high complication rate. The aim of this study was to evaluate whether the implementation of a perioperative care bundle reduced long-term mortality and the Comprehensive Complication Index (CCI) after MAES.

**Methods:**

This study was a single-centre retrospective cohort study. Data in the intervention group were collected prospectively and compared with a historical cohort from the same centre. It includes adult patients undergoing MAES. We implemented a care bundle under the name Abdominal Surgery Acute Protocol (ASAP). We initiated fast-track initiatives and standardised optimised care in before, during and after surgery. Data were analysed using survival analysis and multiple regression.

**Results:**

We included 120 patients in the intervention cohort and 258 in the historical cohort. The one-year mortality rate was 21.7% in the intervention cohort compared to 28.3% in the standard care cohort. Adjusted odds ratio of one-year mortality 0.81 (CI95% 0.41–1.56). The 30-day mortality was lowered from 19.0 to 6.7% (*p* = 0.003). The CCI in the intervention cohort was 8.7 (IQR 0–34) compared to 21 (IQR 0–36) in the control cohort (*p* = 0.932) The length of stay increased by two days (*p* = 0.021). Most cases had 71–80% protocol compliance.

**Conclusion:**

Implementing bundle care in major abdominal emergency surgery lowered the 30-day postoperative mortality. The difference in mortality was preserved over time although not significant after one year. The changes in the Comprehensive Complication Index were not statistically significant.

## Background

Depending on the population, mortality rates range from 3.6 to 48% after major emergency abdominal surgery (MAES) [1–7]. Conditions requiring MAES such as bowel obstruction, perforation or ischaemia are associated with inflammation, sepsis and pain [8–10]. The systemic inflammation or haemodynamic instability in combination with surgical stress results in a risk of organ failure and postoperative complications [11, 12]. Optimising the care of patients undergoing MAES has received increased attention [13–18]. Previous attempts at optimising perioperative initiatives for patients undergoing MAES have shown a decrease in mortality after implementing quality improvement programmes by reducing mortality from 21.8% to 15.5% and from 14% to 10.5%, respectively [13, 14]. However, the most extensive study in this area found no effect of implementing a large-scale multicentre optimising protocol [17].

Complications influence the postoperative recovery [19]. The Clavien–Dindo score only accounts for individual complications, but the more recent comprehensive complication index (CCI) includes *all* complications over a course of time to form a single score. None of the prior studies investigated the influence of a quality improvement programme on one-year mortality or the CCI [20–22]. This study aimed to evaluate the effect of a multidisciplinary perioperative bundle on 30-day mortality, one-year mortality and the postoperative CCI.

## Methods

### Study design

This study was a retrospective cohort study on a process improvement project. Data were prospectively collected in the intervention group. The intervention was a multidisciplinary perioperative bundle. A historical consecutive control cohort from before the bundle was used for comparison. No approval was needed from the ethical board for this type of study.

### Setting

A multidisciplinary bundle named Abdominal Surgery Acute Protocol (ASAP) was implemented and covered the entire perioperative course from admission to discharge. Data for the control cohort were collected over a two-year period from January 2015. For the intervention cohort, data were consecutively collected from June 2018 until June 2019. Data were gathered from a single hospital with a patient base of 350.000 people. Patients were initially admitted to an emergency department with abdominal surgeons present. Postoperative complications were registered up to 30 days after surgery. Mortality was registered up to one year after surgery.

### Participants

Patients were included if they had MAES. Patients who had surgery for a complication through elective surgery were excluded. Patients transferred from other wards or hospitals were also excluded. MAES was defined as either laparotomy or laparoscopy due to emergency abdominal pathophysiology that did not meet the exclusion criteria. The exclusion criteria included surgery due to trauma, appendicitis or cholecystitis. The following conditions were included in the intervention cohort: surgery due to a bowel obstruction, intestinal ischaemia, intestinal perforation, intraabdominal abscess and intraabdominal bleeding requiring surgical management.

### Implementation

Before implementation, the ASAP was presented to the entire staff of in-house doctors. Doctors and nurses in the emergency department were informed about the ASAP contents and directed to inform surgeons immediately if they suspected a MAES patient. Surgeons and nurses had multiple teaching sessions in both the emergency and the surgical departments. Anaesthetists, nurse anaesthetists, surgical nurses and emergency nurses received a verbal presentation before the implementation started. All doctors were given a pocket card describing the content of the ASAP, which was specific for their area of speciality. During the study period, surgeons received teaching sessions every other month. All other departments had two or more lectures to ensure compliance and information for new staff. As data were monitored during the study, staff were presented with an audit after three months. To monitor implementation, 12 key elements were tracked.

### Interventions

**Fig. 1:**
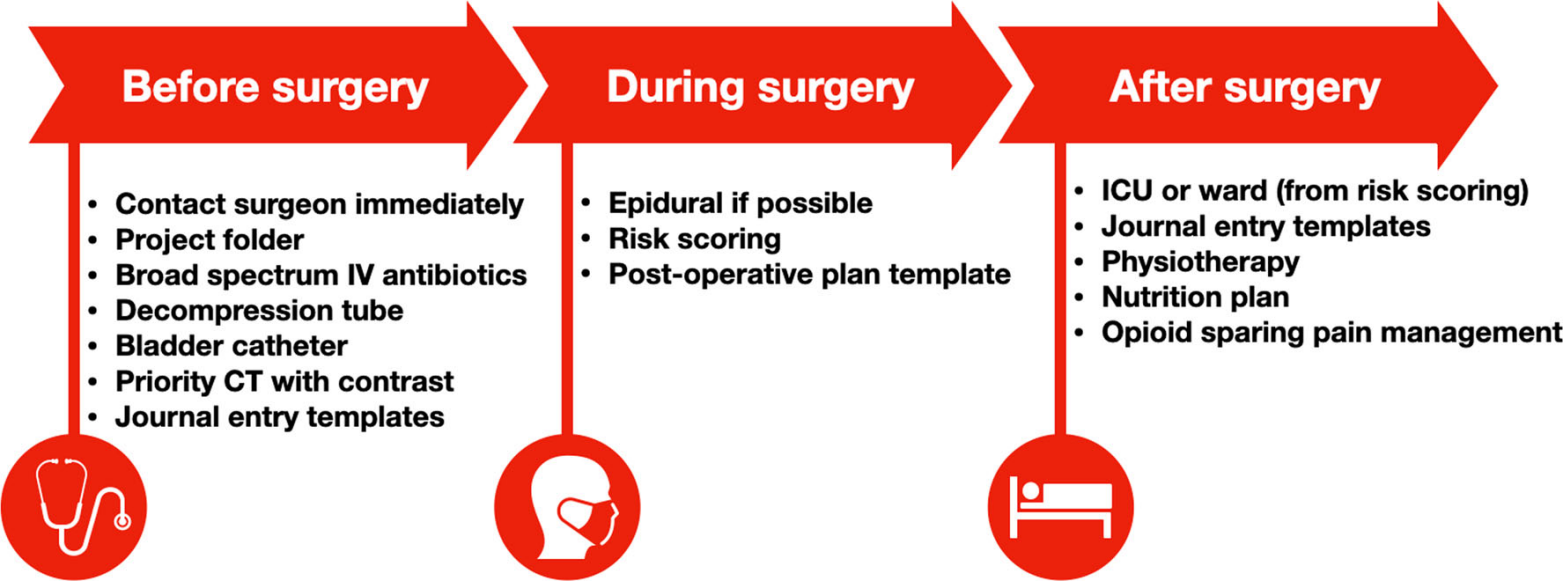
Elements of the abdominal surgery acute protocol

The ASAP consisted of both clinical and administrative actions. The elements in the ASAP were selected from prior research and expert opinions. The elements were finally chosen for implementation if implementation was feasible. The contents of the ASAP are summarised in Fig. 1.

### Preoperative initiatives

Broad-spectrum intravenous antibiotics were started before diagnosis and work-up to ensure that treatment for possible septic or preseptic conditions was not delayed [23]. Antibiotics were preceded by blood culture. A nasogastric decompression tube was administered to avoid further intestinal loading and vomiting. A urinary catheter was administered to monitor urinary output and kidney function. A fast-track abdominal CT scan with IV contrast was prioritised by the radiology department and was performed within one hour to enable fast and correct diagnosis before possible surgery [24, 25]. Specially designed templates were used in patient health records as an entry note for the ASAP and as a referral for CT and surgery. Templates were utilised to ensure uniformity and correct documentation. Patient's information was added to a project folder containing checklists and timestamps. If the wait from a CT scan would be a deteriorating factor, this step was bypassed and the patient was scheduled for immediate surgery. The maximum waiting time from deciding to operate until starting the surgery was set as six hours to avoid deterioration of the patients [26–28]. All decisions following initialisation were made by specialist registrars, senior registrars or consultants. Before surgery all patients were evaluated by a consultant anaesthesiologist and prioritised for a dedicated operating room.

### Intraoperative initiatives

If there were no contraindications, patients were administered a thoracic epidural to ensure sufficient analgesia in the postoperative period while lowering the use of opioids [29]. At the end of the surgery, risk scoring was performed using the surgical APGAR score [30]. Lower surgical APGAR score is associated with increased postoperative mortality and admission to intensive care [30, 31]. If the surgical APGAR was ≤4 or the ASA score > 2, the patient was categorised as a major risk patient, otherwise as minor risk patient. Major risk patients were transferred to either the intensive care unit or the intermediate care unit immediately after surgery and remained there for at least 24 h. Minor risk patients were transferred to the ward after at least eight hours of observation in the recovery unit. The triage of patients to intensive care was selected to ensure intensified care only to patients who were at greater risk of postoperative mortality, while not flooding the intensive care unit with patients who were not. The postoperative plan was documented in a specially designed template within the patient’s health records.

### Postoperative initiatives

In case of confirmed intraabdominal sepsis, we continued intravenously administered antibiotics. Antibiotics were given for at least three days and then evaluated daily. Rounds were documented using a template covering pulmonary status, mobility, bowel function, fluids, nutrition, infections and overall clinical assessment. To avoid pulmonary complications and delayed wound healing, physiotherapy took place as soon as possible following surgery [32, 33]. The patient’s nutrition state was evaluated daily from the first postoperative day, and patients unable to sustain nutritional need orally were supplemented with nutrition by feeding tube or parenteral nutrition. We used an opioid sparing regime where patients with epidural analgesia were only given morphine if needed. In cases without epidural analgesia, patients were given prolonged release tramadol twice daily.

### Variables

The primary outcome was one-year mortality. The secondary outcomes were the CCI and 30-day mortality. Exposure was inclusion in the ASAP protocol. The CCI summarises all postoperative complications, creating one score for each patient based on the Clavien–Dindo scores. Once calculated, the index went from zero to 100, with zero being no postoperative complications and a score of 100 being death from a complication [22]. Follow-up was carried out by investigating mortality up to a year after surgery.

### Data sources

Data in both the intervention cohort and the control cohort were gathered from the electronic patient files.

### Bias

Complications were noted in the electronic health records by abdominal surgeons and medical doctors during rounds. The investigators were limited to reading journal entries, CT descriptions, surgery notes, etc. This meant that if a complication was not in the patient file, it was not included in the study.

### Study size

All patients undergoing MAES were included in the ASAP. For this study, patients who were transported from other hospitals or wards were excluded. Patients were also excluded if they had MAES due to a complication from elective surgical procedures.

### Quantitative variables

BMI was grouped as either underweight (<18.5), normal weight (≥18.5) or overweight (≥ 25) [34]. The Charlson Comorbidity Index was grouped as mild (0–2), moderate (3–5) and severe (>5) [35]. The degree of intraabdominal contamination was registered as either none/localised or diffuse. We monitored 12 protocol elements to evaluate to implementation of the ASAP. The monitored elements were; The ASAP folder, initial assessment template, preoperative antibiotics, preoperative decompression tube, CT scan within one hour, surgery booking template, surgery within 6 h from decision to operate, epidural analgesia when eligible, postoperative plan template, arrival note, postoperative physiotherapy and rounds template.

### Statistical methods

For comparing continuous variables, we used the Kruskal–Wallis test since age and duration of surgery were not normally distributed. For the categorical variables we used the *X*^2^ test to compare the ASAP group with the standard care group. Continuous variables were reported as median + interquartile range. Categorical variables were reported as number and per cent). Multivariate logistic regression was used to investigate the reduction in the mortality risk. The regression analyses were adjusted for baseline demographics (age, sex, the Charlson Comorbidity Index group and BMI group). Furthermore, we adjusted for surgical diagnosis and degree of intraabdominal contamination as some diagnoses have higher mortality rates [36], and intraabdominal contamination increases the risk of postoperative sepsis [37]. Overall survival analysis was estimated by the Kaplan–Meier method and compared using the log-rank test. Patients with missing data were firstly noted and secondly omitted from the regression analysis. There was no loss when it came to follow-up in terms of mortality. Statistical significance was set to *p* <0.05 or confidence interval not containing 1.0.

## Results

**Fig. 2:**
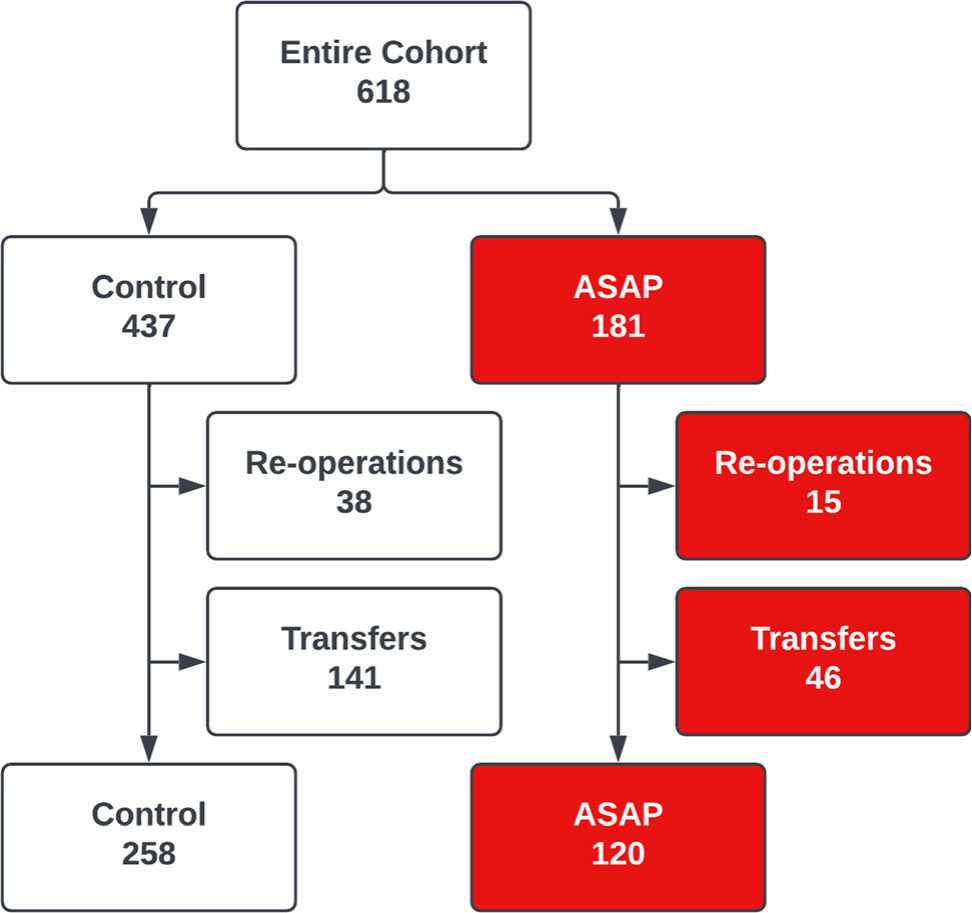
Entire cohort: 618 patients. Exclusions: Re-operations (Controls: 38, Protocol: 15); Transfers (Controls: 141, Protocol 46). After excluding re-operations and transferred patients, 120 patients remained in the protocol cohort and 258 patients in the control cohort

During the study period, 618 cases were classified as MAES, including both the historical and prospective cohorts. After excluding re-operations and transferred patients, 120 patients remained in the ASAP cohort and 258 patients in the control cohort (Fig. 2). The two groups were comparable in all demographic variables except for tobacco use and diagnosis of ischaemia/necrosis (Table 1).

**Table 1:** Characteristics of both ASAP and control cohort

Variable	ASAP	Control	*p*-value
Age	71 (58–78)	69 (61–78)	0.703
Male	51 (42.5)	124 (48.1)	0.369
Smoker	22 (19.6)	85 (35.0)	0.005
Diabetes	17 (14.3)	36 (14.6)	1.000
ASA > 2	48 (26.5)	63 (34.8)	0.111
Surgery duration	113 (68–171)	102 (67–149)	0.304
Open surgery	90 (75.0)	177 (68.6)	0.250
*BMI*			
Underweight (< 18.5)	7 (6.0)	20 (9.9)	0.453
Normal weight (18.5–25)	56 (47.9)	89 (44.1)	
Overweight (> 25)	54 (46.2)	93 (46.0)	
*Medication*			
Steroid use	7 (6.0)	19 (7.7)	0.701
Blood thinner	34 (29.1)	76 (30.9)	0.816
Statins	21 (17.9)	53 (21.6)	0.501
*Charlson Comorbidity Index*			
Mild (0–2)	32 (26.7)	81 (31.4)	0.349
Moderate (3–5)	60 (50.0)	132 (51.2)	
Severe (> 5)	28 (23.3)	45 (17.4)	
*Surgical diagnosis*			
Mechanical obstruction	79 (65.8)	146 (56.6)	0.111
Intestinal perforation	27 (22.5)	49 (19.0)	0.513
Perforated ulcer	9 (7.5)	33 (12.8)	0.178
Ischaemia/necrosis	3 (2.5)	24 (9.3)	0.030
Intraabdominal bleeding	1 (0.8)	2 (0.8)	1.000
Intraabdominal abscess	1 (0.8)	4 (1.6)	0.938

*n*(%). Age and duration of surgery stated as median and IQR. Missing values: Smoker (ASAP: 8, control: 15)—Diabetes (ASAP: 1, control: 12)—BMI (ASAP: 3, control: 56)—Surgery duration (ASAP: 0, control: 84)—Steroid use, anticoagulants and statins (Intervention: 3, control: 12)

### Mortality

**Fig. 3:**
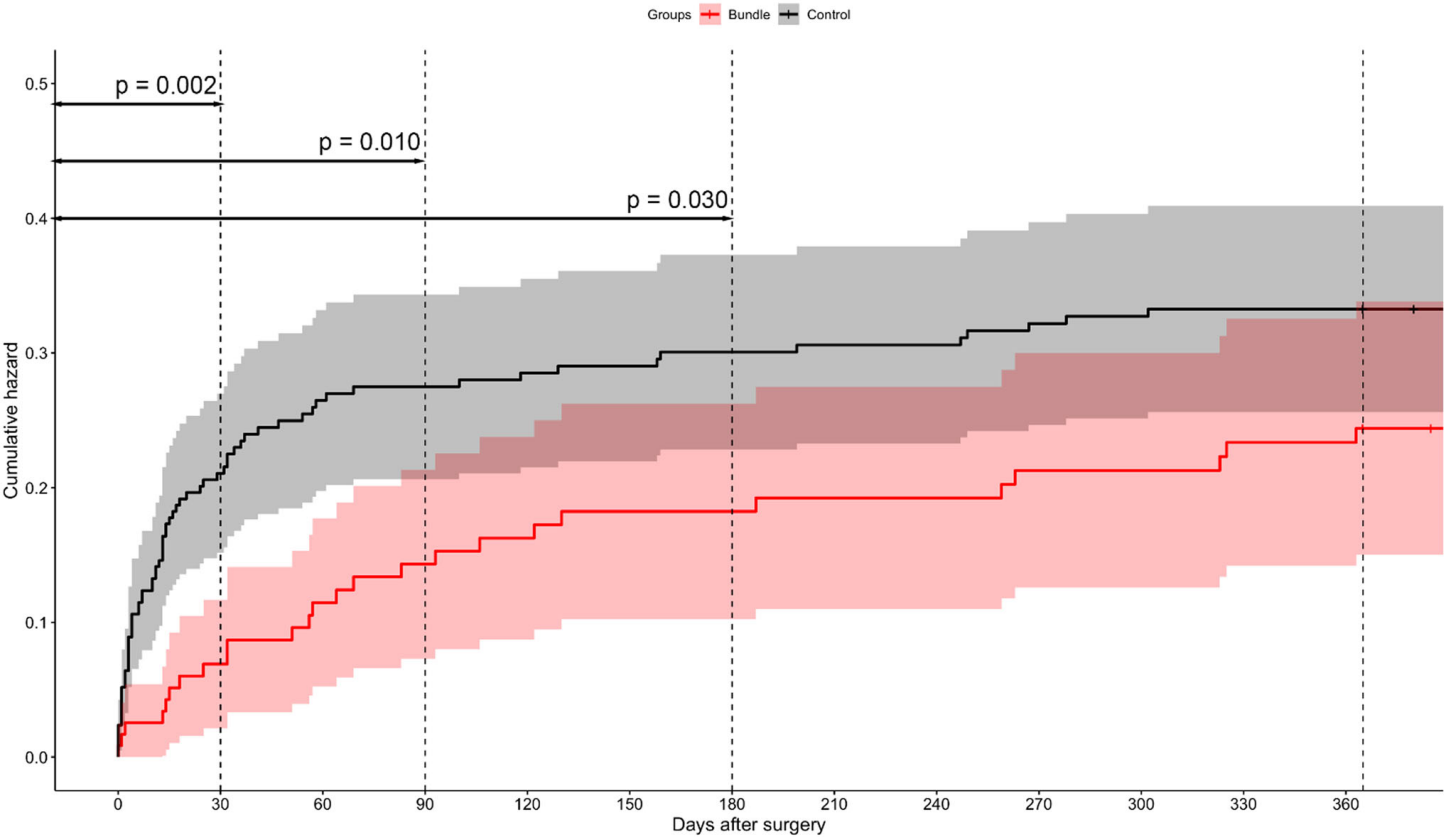
Survival analysis, including confidence intervals, up to 365 days after surgery. Red line = ASAP group, Black line = Control group. Dotted lines at 30, 90, 180 and 365 days. Log-rank *p*-values: 30 days (*p* = 0.002), 90 days (*p* = 0.01), 180 days (*p* = 0.03), 365 days (*p* = 0.1)

Crude one-year mortality was 28.3% in the standard care cohort and 21.7% in the intervention cohort (*p* = 0.215). The 30-day mortality was lowered from 19.0 to 6.7% (*p* = 0.003), which equals a relative risk reduction of 64.7%. The unadjusted survival analysis found an overall *p*-value of 0.1 for one-year mortality follow-ups. For 30 days, the *p*-value was 0.002 (Fig. 3). Adjusted logistic regression found that the ASAP did not reduce the risk of mortality at one year (OR 0.81; CI95% 0.41–1.56). However, the adjusted regression found a reduced risk of 30-day mortality (OR 0.31; CI95% 0.1–0.84) (Table 2).

**Table 2:** Adjusted logistic regression on both 30- and 365-day mortality

	30-day mortality			One-year mortality		
	OR	95 CI	*p* value	OR	95 CI	*p*-value
ASAP	0.31	0.10–0.84	0.027	0.81	0.41–1.56	0.526
age	1.00	0.96–1.05	0.867	1.04	1.00–1.07	0.042
Female	1.84	0.71–5.06	0.219	0.82	0.42–1.58	0.552
Smoker	0.27	0.07–0.87	0.038	1.10	0.50–2.38	0.805
Charlson Moderate (3–5)	4.82	0.62–105.61	0.193	2.78	0.82–11.5	0.123
Charlson Severe (> 5)	60.6	7.35–1417.9	0.001	10.02	2.74–44.5	0.001
Localised contamination	1.94	0.29–10.84	0.467	1.17	0.30–4.23	0.811
Diffuse contamination	4.07	0.94–19.13	0.065	3.39	1.07–11.4	0.042
Mechanical obstruction	1.41	0.10–41.29	0.810	2.37	0.26–54.5	0.492
Intestinal perforation	0.66	0.05–18.00	0.767	1.11	0.12–24.5	0.935
Perforated ulcer	3.05	0.23–88.02	0.434	1.44	0.14–33.8	0.777
Ischaemia/necrosis	13.19	0.78–455.35	0.096	3.74	0.32–97.5	0.334
Alcohol overuse	5.09	1.04–23.48	0.037	1.00	0.28–3.22	0.999
Underweight	2.38	0.58–9.17	0.213	3.72	1.37–10.4	0.011
Overweight	0.38	0.13–1.02	0.063	0.43	0.21–0.85	0.018

The top row shows the adjusted OR of mortality when affected by the protocol

### Complications

**Fig. 4:**
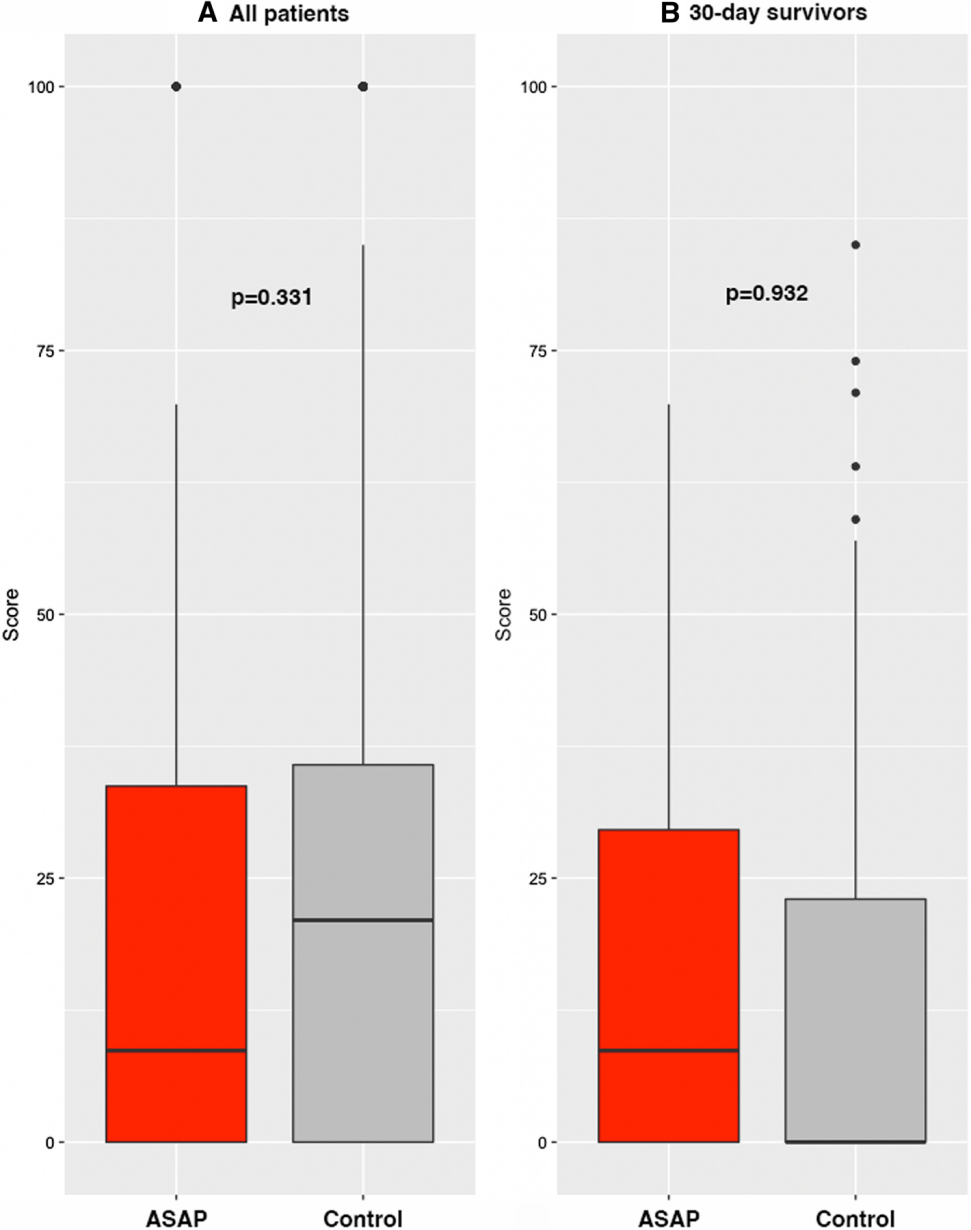
Boxplots showing the distribution of the comprehensive complication index. The median values are represented by the black line. Part **A** shows all patients, and part **B** shows only patients that survived the first 30 postoperative days. All patients (*p* = 0.331), 30-day survivors (*p* = 0.932)

For all complications (Clavien–Dindo 1–5) the rate was similar for the two groups (56.7% for the optimised patients and 56.2% for the control group). The rate of complications with a Clavien–Dindo score >2 was 25.8 vs 30.6% for the control group (*p* = 0.405). The total CCI was 8.7 (IQR 0–34) for the ASAP group vs 21 (IQR 0–36) for the control group, *p* = 0.331. The CCI for 30-day survivors was 8.7 (IQR 0–30) in the ASAP group vs 0 (IQR 0–23) in the control group (*p* = 0.032). The distribution of CCI is displayed in Fig. 4. There were 4 (4.2%) unplanned admissions to the intensive care unit in the ASAP group compared with 22 (8.5%) in the control group (*p* = 0.188). In the ASAP group, one patient was admitted to ICU due to sepsis compared with nine patients in the ASAP group. The length of stay was increased in the ASAP group compared to the control group (5 vs 7 days (*p* = 0.021)).

### Implementation

**Fig. 5:**
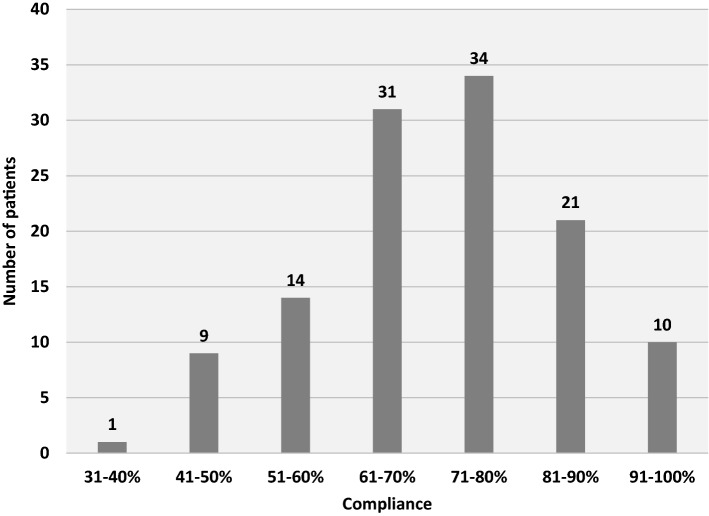
Percentage compliance to monitored protocol elements shown in steps of 10

Most of the patients in the ASAP group had 71–80% compliance of protocol elements, and one patient had < 40% compliance. The distribution on protocol compliance is shown in Fig. 5. Protocol compliance at patient level is included in “Appendix 1”.

## Discussion

Implementing a multidisciplinary perioperative bundle in major abdominal emergency surgery decreased the postoperative mortality. We were not able to detect statistically significant changes in the CCI.

This is the first study investigating one-year mortality and using the CCI after the implementation of an optimising bundle in MAES. Throughout the observation period, the ASAP bundle patients had lower mortality. However, the difference was most noticeable in the short-term postoperative period, and we did not find significant improvement in mortality after one year. An earlier study showed a significant decrease in 180-day mortality from 29.5 to 22.2 [13], which is in line with our findings. At 30 days, we found a crude mortality of 6.7 vs 19%. This risk reduction was more pronounced than reported earlier in both the AHA study and the ELPQuiC study [13, 14]. In the AHA study, 30-day mortality was lowered from 21.8 to 15.5% and in the ELPQuiC study, from 14.0 to 10.5%. When comparing our results with these studies, we found both similarities and differences. The ELPQuiC study included patients undergoing laparotomy, whereas our bundle and the AHA study also included laparoscopies. The AHA study had more cases with a perforated viscus compared to our study and as this is a severe condition, it could explain why the AHA did not achieve a lower postoperative mortality rate. The patients' age in our study was slightly higher than in the AHA study and higher than in the ELPQuiC study. However, the number of patients with an ASA score > 2 was lower in our study. The lower ASA score in our patients could potentially have an impact on mortality. The AHA study was similar to the ASAP in implementing elements in both the pre-, intra- and postoperative course, whereas the ELPQuiC did not implement beyond the initial postoperative destination.

Major risk patients were transferred to either intermediate care unit or intensive care unit after surgery. The AHA study utilised the recovery unit for 24 h and the ELPQuiC study transferred *all* patients to the intensive care unit for postoperative care. The initial postoperative period can be critical, and our bundle allowed us to differentiate between cases.

Our study utilised entry-note templates for all patients, making sure that care was both optimised and uniform. Neither the AHA nor the ELPQuiC study used this approach, and we believe that this was an important step to increase the attention given to the patients.

The ASAP did not improve complication rates, and the differences in CCI between groups were not statistically significant. However, the CCI might be lower in the ASAP group. Within 30-day survivors, the CCI might be higher in the ASAP group. This was most likely due to a decreased failure to rescue, meaning that the ASAP might have allowed patients to survive worse complications [38]. This would have led to a higher CCI without subsequent mortality. The AHA study reported fewer patients with major complications (Clavien-Dino score >2). We saw the same tendency concerning major complications. However, this was not significant. The length of stay increased by two days after implementing the ASAP and we attribute this to a decrease in failure to rescue. When patients survive worse complications, it might cause longer stays. The AHA study also found an increased length of stay. The EPOCH trial was a large-scale stepped-wedge cluster-randomised trial, including 93 hospitals [17]. Each hospital had a taskforce that selected points of care to use from the EPOCH bundle. This makes it difficult to draw a comparison with our study. Nonetheless, the EPOCH trial was not successful in lowering mortality.

We did not demonstrate a statistically significant reduction in one-year mortality. The mortality analysis was adjusted for age, sex and Charlson Comorbidity Index which dimmish the influence these factors have on the results. It could be that MAES does not impact the mortality further after a certain amount of time or that the effect of the ASAP diminishes over time. When performing multivariate regression, a similar pattern concerning significance was found where the risk reduction was significant at 30 days. The difference in CCI was not significant and this was possibly due to the distribution of the index. This was a quality improvement project and numbers were limited by availability. A post hoc analysis based on the differences seen in this study suggests that over 1,000 patients would be needed in each arm to achieve statistical significance.

There was a risk of a detection bias, given that potentially missed cases could have influenced the differences between our cohorts. However, the inclusion period for the historical cohort is twice of the prospective cohort, which corresponds with the number of patients. As this was a non-blinded study, there was also a risk of observer bias.

The degree of protocol compliance could also have affected the results. As shown in Fig. 5, most cases had more than 60% compliance to the protocol. However, a study performed to analyse the impact of protocol compliance in the ASAP revealed no benefit from high compliance vs low compliance [39]. Furthermore, the study found that more than half of the elements were successfully implemented with the element being used in more than 70% of the cases.

After implementation of this bundle, it remains the standard in our surgical emergency service. The continued efforts in major abdominal emergency surgery are important. Additional studies regarding protocol compliance could be beneficial to evaluate whether some elements are more important than others in patients who are already optimised.

## Conclusions

Using bundle care in major abdominal emergency surgery lowered the 30-day postoperative mortality. The difference in mortality was preserved over time although not significant after one year. The changes in the Comprehensive Complication Index were not statistically significant.

## Data Availability

Anonymised data will be made available upon request.
